# Editorial: Aging and chronic disease: public health challenge and education reform

**DOI:** 10.3389/fpubh.2023.1175898

**Published:** 2023-05-09

**Authors:** Xiaodong Sun, Xuan Li

**Affiliations:** ^1^Department of Endocrinology and Metabolism, Affiliated Hospital of Weifang Medical University, Weifang, China; ^2^Clinical Research Center, Affiliated Hospital of Weifang Medical University, Weifang, China; ^3^Department of Physiology and Biophysics, University of Mississippi Medical Center, Jackson, MS, United States

**Keywords:** aging, chronic disease, public health, education, health care

The aging of the global population has led to significant changes in its age structure, as life expectancy has increased while fertility rates have declined (1, 2). This demographic shift has given rise to major concerns worldwide, particularly in developing countries, where the growing number and percentage of older adults have become a pressing issue (3, 4). In addition, the aging population has resulted in a rise in chronic diseases such as diabetes, hypertension, and obesity, as well as various mental health issues such as depression, which pose serious public health problems (5, 6). The resulting burden on individuals, families, and society in terms of rehabilitation, stress, and financial strain is substantial, highlighting the need for healthcare and education reforms to promote healthy aging (7). Thus, health care or health-related education reform is needed to address the issues associated with this aging trend and achieve healthy aging. This research special issue provides a platform for the latest advances in a range of issues and strategies to address the problems posed by population aging and chronic disease. The special issue collects 34 original research articles, one review article, and one opinion article.

The high prevalence of chronic diseases such as obesity, hypertension, diabetes, and metabolic syndrome is a significant public health concern, particularly among the older adults. Various indicators of obesity have been identified to reflect obesity under different conditions. Using the Chinese Family Panel Studies 2018 panel data, Wang et al. found that body mass index may be influenced by interactions between lifestyle variables and educational achievement. Additionally, a community-based cross-sectional study conducted by Fan et al. revealed novel obesity-related indicators, including relative fat mass and lipid accumulation product, which were previously unreported to have adverse effects on health-related quality of life. In another study, Du et al. found that body fat percentage was positively associated with the risk of H-type hypertension (homocysteine levels ≥10 mmol/L) in postmenopausal women.

Metabolic diseases pose a significant burden on older individuals and increase the risk of cardiovascular disease. An original research study by Xing et al. observed that the detection rate of people at high risk of cardiovascular disease was high in Anhui Province, China (21.46%). Cardiomyopathy and myocarditis are common cardiovascular diseases that can lead to heart failure in aging adults, as observed by Zhang and Cheng et al. using the Global Burden of Disease 2019 data. High systolic blood pressure and alcohol consumption are among the top risk factors for these serious cardiovascular diseases worldwide. Thus, preventing cardiovascular disease become more critical. For example, tooth extraction is common but has cardiovascular responses in aging adults. Li et al. demonstrated that older adults had a significant cardiovascular response during tooth extraction. This highlighted the critical role of monitoring heart rates and blood pressure during tooth extraction.

Chronic kidney disease is another chronic disease that is associated with several adverse events. Huang et al. and Song et al. identified an association between chronic kidney disease and cataract and sarcopenia prevalence, respectively. In addition, Zhang Z. et al. concluded that serum soluble Klotho levels were positively correlated with estimated glomerular filtration rate and inversely correlated with chronic kidney disease stages, particularly in older patients with obesity and diabetes.

Interestingly, several potential risk factors for older individuals with chronic diseases display gender-specific patterns. Wu et al. have identified potential causal correlations between metabolic syndrome and elevated total bilirubin levels in females, as well as links between a rise in urea levels and an improved fatty liver disease index in males. Similarly, Sun and Lu et al. have discovered that factors influencing health literacy were associated with gender differences in older patients with chronic diseases. Additionally, in a cross-sectional study, Zeng X. et al. found that aging individuals with diabetes face an increased risk of frailty and pre-frailty (22.7 and 58.5%, respectively) associated with living alone, low income, and multimorbidity. Besides metabolic disease, cancer is also a prevalent chronic disease in the aging population. This is supported by analyzing the characteristics of China's cancer epidemic over 12 years. Guo et al. have noted that while cancer incidence in Chinese adults aged 60 and above has shown a declining trend, it remains significantly high.

In addition to physical health, it is crucial to emphasize the importance of addressing mental disorders such as depression, cognitive impairment, and dementia in aging adults. Late-life depression is a prevalent mental illness with devastating effects on the older adults. Morita et al. conducted a study on intimate social networks among 660 community-dwelling older persons and found that aging adults tended to bond with others with similar depressive symptoms. They also identified significant apathy homophily but no significant suicidal ideation homophily. Cognitive impairment can exacerbate various comorbidities, as shown in a cross-sectional study by Vera et al., which favored the clustering of three comorbidity patterns: (1) arthritis, asthma, respiratory diseases, and depression; (2) obesity, diabetes, hypertension, and hypercholesterolemia; (3) heart attack, coronary heart disease, stroke, and kidney disease. Thus, a multidisciplinary approach should be taken to treat patients with “perceived cognitive impairment.” Furthermore, in a longitudinal population-based study, Xu et al. examined the relationship between participation in leisure activities and recovery from mild cognitive impairment in older adults. They found that participants with the highest leisure activity engagement had the greatest chance of mild cognitive impairment reversion. Finally, dementia is a degenerative condition that primarily affects older adults and negatively impacts their quality of life. Dong et al. studied the risk of falls at home in Chinese people with dementia and concluded that aging adults with a history of falls were at greater risk of falling, and assessing risk factors could prevent dementia. Lu Y. et al. highlighted the importance of proactive community-based surveys that could increase the early detection of dementia.

With the increasing aging population, chronic diseases contribute significantly to the cohort trends in disability. This is evident from a longitudinal study by Pan et al., which analyzed five-wave national representative data to examine the incidence of disability in older age groups in China. The study found that both age and cohort trends indicate a higher likelihood of disability. As a result, society must focus more on preventing disability in aging adults to improve their quality of life.

In promoting healthy aging, the rational use of medicines is crucial. Lu V. et al. conducted a three-year cross-sectional study showing that inappropriate drugs were commonly used in older adults with diabetes. The study identified clinical comorbidities such as chronic gastrointestinal disease, osteoarthritis and rheumatoid arthritis that increase the likelihood of potentially inappropriate drug exposure. Therefore, minimizing the risk for these patients requires considering their clinical comorbidities. In another study, Eschbach et al. argued that substance abuse is a public health crisis in the United States and described an approach that states can use to coordinate state and federal funding to prevent opioid misuse. Community-based education is a way to improve health and reduce disability and death associated with substance abuse. Ferro-Uriguen et al. explored the pharmacotherapeutic outcomes of a person-centered prescribing intervention in frail older adults. The study found that this intervention reduced medication therapy costs, improved medication metrics, and decreased the total amount of medication routinely administered to older adults. These findings underscore the importance of rational use of medicines in promoting healthy aging.

In order to prevent and mitigate inappropriate medication use among aging adults, promoting medication literacy is of paramount importance. A cross-sectional, observational study conducted by Shen et al. examined the intersection between medication literacy and social support in older adults with hypertension. The findings of this study suggest that social support plays a crucial role in promoting medication knowledge among this population. Furthermore, Tang et al. designed, implemented, and evaluated a medication management model that leverages the services of primary care physicians. Ultimately, the model was found to reduce the number of multimorbidity medications in older adults and improve medication safety and adherence.

In the era of globalization and diversification, migration has become a widespread phenomenon, and older migrants are particularly vulnerable to health problems. Zhong et al. conducted a study to assess the association between migration and hospitalization and found that older migrants in urban areas after retirement are more likely to require hospitalization, especially those with chronic illnesses. To address this disparity in healthcare utilization, providing high-quality chronic illness management and early intervention to this population is crucial. Additionally, public health education must be promoted to improve the health status of older migrants, as demonstrated by the studies conducted by Yan et al. and Zeng W. et al.. These studies highlight the importance of developing and implementing appropriate teaching strategies and standardizing public health education to meet the unique characteristics and preferences of older migrant populations. Overall, these findings emphasize the need for a comprehensive approach to address the health challenges faced by older migrants in a globalized and diverse world.

The global increase in life expectancy and changes in the health status of the aging population have highlighted the crucial need for professional rehabilitation and therapists. In a study by Sun and Lin et al., the quality and model of rehabilitation therapy education in China were evaluated, and it was suggested that national policies should accelerate rehabilitation education and optimize rehabilitation curriculum to improve the quality of rehabilitation services. Bonnechère et al. argued that technology-assisted interventions should be employed in low- and middle-income countries to increase the quality of rehabilitation services since they are critical in managing chronic disorders. Accordingly, Jing et al. proposed that employing more rehabilitation therapists is necessary to provide high-quality therapy since most therapists work under severe pressure. Moreover, staff satisfaction is also vital. In a cross-sectional survey, Cai et al. emphasized the need to enhance primary healthcare professionals' salary, improve their material circumstances, and attend to their self-improvement demands.

In addition to rehabilitation therapy, other approaches can help achieve this goal. Yuan et al. suggest that telecare services may be a viable alternative for empty-nesters with chronic illnesses. Meanwhile, Zhang P. et al. found that health shocks can lead to positive changes in preventative behaviors, such as reducing the likelihood of smoking and alcohol consumption and increasing the likelihood of undergoing auxiliary inspections during physical exams. The role of older adult education in healthy aging was also reviewed by Zhang and Kan et al.. While older adult education can play an important role, its limitations must be recognized, and strategies should be developed to promote active aging.

Despite these efforts, some issues persist. For instance, many patients turn to online sources for medical advice and education. However, the quality and reliability of medical information on websites like YouTube can be questionable, as found by Zhang X. et al.. To address this, academic organizations should focus on producing high-quality video content and marketing it effectively to reach more viewers. Furthermore, Podhorecka et al. suggested healthcare workers must establish appropriate contact with older people, and medical education should be continuously updated and improved.

Overall, the articles in this Research Topic highlight the challenges of healthy aging and suggest measures to address them. Further research is necessary to explore these issues and develop effective solutions for the aging population.

## Author contributions

Both authors listed have made a substantial, direct, and intellectual contribution to the work and approved it for publication.
